# New Morphological, Distribution, and Ecological Data on *Scabiosa garganica* (Caprifoliaceae), a Poorly Known Species of the Italian Flora, with Evaluation of Its Conservation Status and Typification of the Name

**DOI:** 10.3390/plants12091915

**Published:** 2023-05-08

**Authors:** Daniele Bonsanto, Nello Biscotti, Robert Philipp Wagensommer

**Affiliations:** 1 Department of Agricultural, Food and Environmental Sciences (D3A), Marche Polytechnic University, I-60131 Ancona, Italy; 2 Faculty of Education, Free University of Bozen-Bolzano, I-39042 Brixen-Bressanone, Italy

**Keywords:** ecology, Italy, IUCN, lectotype, morphology, taxonomy

## Abstract

This paper presents the results of a research performed on Gargano Promontory (SE-Italy) on the populations of *Scabiosa garganica*, a species with little herbarium records and whose few morphological descriptions are outdated. *S. garganica* belongs to the *S. holosericea* aggr., a group including very similar taxa that still have different taxonomic classifications. Its typical location is Monte Sant’Angelo in the Gargano area. Surveys have ascertained the existence of many populations, whose stational data help to understand the distribution and ecological conditions *S. garganica* is linked to. The morphological analysis of a large sample (75 plants from 9 sites) allows for the description of the qualitative and quantitative characteristics of this species. The new morphological framework highlights the species autonomy of *S. garganica* and can contribute to clarifying the relationship with *S. holosericea* and *S. taygetea* to which it is closer. In addition, for the correct delimitation of the species, the name *Scabiosa garganica* is lectotypified. Finally, the species was assessed against the IUCN criteria for the evaluation of its conservation status.

## 1. Introduction

This research focuses on *Scabiosa garganica* Porta & Rigo ex Wettst. [*=S. taygetea* Boiss. & Heldr. subsp. *garganica* (Porta & Rigo ex Wettst.) Hayek] (Caprifoliaceae), a species alternatively considered endemic to Italy [1], occurring in Italy and Greece [2], or as a synonym of *S. taygetea* in Albania [3]. In Italy, it is known to grow with certainty only in the Gargano area (Apulia) [4]. This species belongs to an extremely polymorphous group (*Scabiosa holosericea* aggr.), thus having a still temporary taxonomic classification [1] that is also considered still unresolved [5]. The species was described on the basis of a collection of Porta and Rigo [6], whose tag says “near Monte Sant’Angelo” in the Gargano area. Actually, a “*Scabiosa garganica*” had been reported a century earlier in southern Gargano by Micheli [7], but unexpectedly it was not included in the Catalogus plantarum Horti Caesarei Florentini [8], where several other plants from Gargano are listed. Some years later, *Scabiosa garganica* was mentioned by Tilli [9], describing it as “*frutescens, villosa et incana, foliis laciniatis, flore ex caeruleo purpuracente*” (shrublike, hairy, and grayish, with fringed edges leaves and flowers of a purplish cerulean). After many years, in the spring of 1874 and without making any reference to what Micheli [7] and Tilli [9] had stated, Porta and Rigo [6] report to have found in “Monte Sant’Angelo and not anywhere else”, a *Scabiosa* they state being new, and, they wrote, “we decided to call it ‘garganica’ after its location”. In their second travel in Gargano in 1875 (for more information on the two travels in 1874 and 1875, see [10]), Porta and Rigo [6] provided further details about the location (“*apricis Montis S. Angelo*, *pascuis saxosis*”—sunny Monte Sant’Angelo, in rocky pastures) and the plant’s phenology (“summer flowering”). The species is afterwards reported in Compendio della Flora Italiana [11] with the binomial *Scabiosa garganica* Arc., but in “Flora Italiana” by Parlatore and Caruel [12], it is included as a variety of *Scabiosa pyrenaica* All., together with *S. holosericea* Bertol., whose presence in the Gargano area is reported by Pasquale and Licopoli [13]. However, the authors state that “‘*Scabiosa garganica’* gathered by Porta and Rigo in Gargano area is a very beautiful and more tomentose form”; moreover, this work highlights for the first time its similarity with *S. taygetea* described from Greece [14]. In Wettstein [15], it is accompanied by an illustrated table and described as a species, and new morphological details are provided: “…a wonderful plant…30 to 50 cm high…with a rhizome full of sterile leaves and 1 to 4 flowering stems” (*Rhizoma caespites foliorum steriles et caules floriferos 1–4 edens*). Wettstein too thinks that such characteristics make it undoubtedly close to *S. taygetea* (found in Peloponnese only), but it is “easily recognisable due to its red-violet/purple colour, a shorter corolla and above all its significantly shorter lobes of the calyx”; in addition, it has “protruding, unusually thick shiny white hairs”. In his annotations, Wettstein concludes that “the presence of the same species in Monte Gargano and in the high Albanian mountains is of great geographical interest”. In the first years of the 20th century, the “Herbarstudien” by Huter [16] reports that “nr. 72” (Porta and Rigo’s herbarium sheet) “can only be used as a form of *S. holosericea* Bert.”. Huter too thinks that its main feature are the thick hairs on the leaves (“soft wool, with a colour ranging from ash grey to velvety whitish”); like in *S. holosericea,* the hairs are simple, not branchy, Huter says, and (except for the basal ones) they are “pinnatifid, or lyrate pinnatifid but with a very large terminal lacinia, at times almost round”. In 1921, it is included among the critical and rare species of the Italian flora analyzed by Lacaita [17], who writes as having found it in 1919 in Gargano, “copious in the cliffs of the western limestone ridge, not far from the village but towards Manfredonia, about 450 metres a.s.l.”. According to Lacaita, the woolly appearance of the leaves “is much more pronounced in Gargano samples than in Wettstein’s sketch of the plant in Albania”. Lacaita adds that *S. garganica* has only been found near the village of Monte Sant’Angelo where Porta and Rigo have found and documented it during their two trips in Gargano area. The morphological notes of Lacaita are such that he cannot distinguish it from *S. holosericea*. To resolve his doubt, he concludes that “several samples from the different Gargano’s stations are needed”. Unfortunately, the partial knowledge of the plant remains unchanged in the following years, but *Scabiosa garganica* undergoes continuous reviews that modify its taxonomic status many times. In Fiori’s “Flora Analitica” [18], Porta and Rigo’s “*Scabiosa garganica*” is mentioned as a species [*S. garganica* (Porta) Fiori]. In “Prodromo della Flora Garganica” [19], it is treated as a subspecies of *S. holosericea* (*S. holosericea* Bert. subsp. *garganica* Huter, Porta & Rigo). In this new combination, Fenaroli [19] gathers the different reports of *Scabiosa columbaria* L. var. *holosericea* (Bert.) Fiori by Pasquale and Licopoli (collected in 1872), Martelli (loc. Testa del Gargano, collected in 1893 (FI!)), Fiori (loc. Monte Sant’Angelo, collected in 1898 (FI!)), Trotter and Forti (loc. Manfredonia and Monte Sant’Angelo, collected in 1907 (FI!)), Lacaita (collected in 1919). Fenaroli attributes to this same combination also the samples collected by himself with Grilli (loc. Santuario di Pulsano in 1960 (Herb. Fenaroli, TR)) and by Agostini (“between Monte Sant’Angelo and Mattinata”, in 1961 (Herb. Fenaroli, TR)). According to Fenaroli, in the Gargano area, either *Scabiosa holosericea* and its “*garganica*” subspecies must be present, and they are distinguishable also for their distribution (since the “*garganica*” subspecies was known only in Monte Sant’Angelo’s station). Later, *Scabiosa garganica* was proposed by Zangheri [20] as a subspecies of *Scabiosa vestita* Jord. (*S. vestita* subsp. *garganica* (Porta & Rigo ex Wettst.) Zangh., *comb. inval.*). In “Flora d’Italia” by Pignatti [21], the “Scabiosa of Gargano” is included in the variability of *Scabiosa holosericea*, highlighting the presence in Gargano Promontory of characteristic populations with “thickly woolly leaves with elongated white-greyish hairs”. In this work, Fenaroli’s combination is deemed “illegitimate”, even though it is stressed that “the rank of this taxon needs to be defined through an appropriate research” that has not been carried out. In the first years of 2000, in fact, the main study materials for this plant still are the samples gathered by Porta and Rigo in the Gargano area, and Licht [22] indicates *S. taygetea* subsp. *garganica* as no longer recorded from Gargano area. In the Checklist of the Italian vascular flora by Conti et al. [23], *S. garganica* is considered as a variety of *Scabiosa taygetea* (*Scabiosa taygetea* var. *garganica* (Porta & Rigo ex Wettstein) Hayek). This combination highlights the greater similarities with the populations described for the fir woods on Mount Taygetus in Peloponnese, as Parlatore and Caruel [12] already emphasized. In the more recent publications about the Italian flora, the *Scabiosa* from Gargano area is considered at species rank (*Scabiosa garganica* Porta & Rigo ex Wettstein) [24], or as a subspecies (*Scabiosa tygetea* Boiss. & Heldr. subsp. *garganica* (Porta and Rigo) Hayek) [1,4]; it is reported as such also in the recent “Flora vascolare del Gargano e delle Isole Tremiti” [25]. Pignatti [1] also mentions *S. holosericea* as present in the Gargano region, while Bartolucci et al. [4] consider it as not present in Apulia. Moreover, Pignatti [1] still keeps the awareness of the existence of a little-known group (*holosericea*) whose arrangement is temporary. After more than a century, even now the same gaps pointed out by Lacaita [17] are still the same, due to the complete lack of studies on this species, which would be absolutely needed to clarify the actual distribution in Italy, also given its relationship with *Scabiosa holosericea* and *S. taygetea*, and considering that a report from Basilicata (Monte Alpi), previously attributed to *Scabiosa taygetea* [26], was recently doubtfully referred to *S. garganica* [27]. Monte Sant´Angelo is the reference site including the latest reports [28,29,30]. At the same time, *Scabiosa holosericea* has been nonetheless reported also in southern areas of Gargano Promontory, but with no herbarium material [31]. *Scabiosa holosericea* s. str. is moreover reported in the analytical key by Licht [22]. Licht (Herb. Garg.-MJG, available online at http://jacq.org/#database, accessed on 16 March 2023) also mentions six herbarium sheets of samples of *Scabiosa taygetea* subsp. *garganica* collected in Vallone di Pulsano, Monte Saraceno, Torre Pucci, Monte Spigno, SS 89 (from km 2.2 to km 2.4; at km 10.2). In the light of these reports, the presence in Gargano Promontory of *Scabiosa garganica* is well beyond Monte Sant’Angelo, distinguishing the historical reports from Monte Sant’Angelo [6,17], Vallone di Pulsano [19], and Testa del Gargano [19], from the recent ones (Monte Pucci, Monte Spigno, km 2–10 of SP 89) with herbarium samples (collected in 1986–2006 by Licht) (Table 1). At the same time, reports of *Scabiosa holosericea* from Gargano need to be (re-)considered.

Therefore, the aims of this paper are as follows: (i) the description of the correct morphology of *Scabiosa garganica*; (ii) clarifying the current distribution in Gargano area; (iii) investigating the ecological conditions to which the species is linked to; (iv) clarifying its relationships within the *S. holosericea* aggr.; (v) the evaluation of the conservation status of the species, according to the IUCN categories and criteria [32]; (vi) the typification of the name *Scabiosa garganica* for the correct interpretation of this name, given that it appears to be still untypified [24].

## 2. Materials and Methods

We analyzed 75 individuals of *Scabiosa garganica* collected in autumn 2020 in 9 sites, 5 in the southern area of the Gargano Promontory, and 4 in the northern area of the promontory (Table S1). Every site was georeferenced by a GPS (Garmin Etrex 100, average accuracy of 3 m).

**Figure 1:**
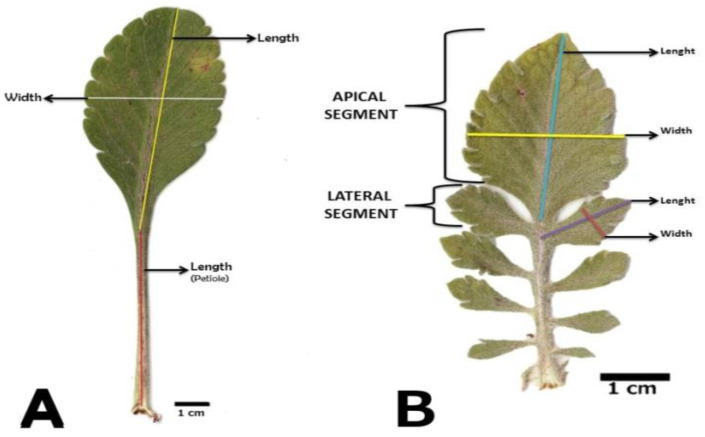
Measurements carried out on leaves: (**A**) basal leaves, (**B**) cauline leaves.

For each individual, morphological qualitative and quantitative characters were analyzed. Leaves (185 basal leaves and 187 cauline leaves) were scanned with a 600 dpi resolution, and following this, they were measured with ImageJ [33] (Figure 1).

*Scabiosa garganica* were compared through the descriptions in the literature [1,6,7,14,15,16,17,21,25] and from the analysis of digitized herbarium sheets of *S. taygetea* and *S. holosericea*, listed in the specimen visa.

For each individual, morphological qualitative and quantitative characters were analyzed.

The measurements carried out were as follows: Plant height, number of scapes, number and diameter of inflorescences, length of flower peduncles, for the basal leaves lamina length and width, length/width, petiole length, and for the cauline leaves total length and width, apical segment (length and width, length/width), lateral segment (length and width, length/width), apical segment width/lateral segment width, and number of lateral lobes. Using SPSS [34], we then performed the standard statistical analysis (mean, standard deviation, min. and max.) of the dataset composed by all measurements, gathered in an Excel spreadsheet (Table S2).

Principal component analysis (PCA), non-metric multidimensional scaling (NMDS), and cluster analysis (CA) using the average linkage method (UPGMA) were performed with PAST package v4.09 software (Natural History Museum, Oslo, Norway) [35]. The similarity matrix was calculated using the Gower coefficient, suitable for mixed data [36].

With regard to the bioclimatic framework, we referenced the recent classification of Pesaresi et al. [37].

The acronyms of the herbaria are according to Thiers [38].

The conservation status of the species was assessed according to the IUCN categories and criteria [32]. The extent of occurrence (EOO) was calculated as convex hull, whereas the area of occupancy (AOO) was calculated using 2 × 2 km grid cells.

## 3. Results

### 3.1. Distribution of Scabiosa garganica

The survey confirmed the presence of many populations both in the northern area of Gargano Promontory (around Peschici) and in the southern area (Monte Sant’Angelo, Manfredonia, Mattinata). In the northern zones, the populations are distributed in a coastal strip going from Torre di Monte Pucci until the last rocky ridges close to Palude di Sfinale, with an altitude range of 20 to 130 m a.s.l. In the southern zones, the populations found are on the other hand distributed in a quite continuous way only in the north-west ridge of Monte Saraceno (Mattinata), and from there alongside the provincial road 55 that leads to Monte Sant’Angelo, with an altitude range between 250 and 670 m a.s.l.

### 3.2. Ecological Characterisation of Scabiosa garganica

The stational data supply useful ecological information: The populations can be found at altitudes ranging between 20 m a.s.l. (Sfinale) and 1000 m a.s.l. (Monte Spigno); the exposure varies from north-west to south-west; and the substrates are rocky dry substrates with flat or even deep soils, often coves of land on small terraces or mountain sides, or at the foot of rocky slopes. The bioclimatic framework [37] belongs to the Mediterranean macro-bioclimate, pluviseasonal-oceanic bioclimate, and lower mesomediterranean thermotype with lower subhumid ombrotype. With regard to the vegetation, both northern and southern areas are dominated by dry rocky *Pinus halepensis* Mill. woodlands, where the populations of *Scabiosa garganica* are linked to clearings or can be found at the borders of the forest. An important aspect is that the species can be found mostly in areas recovering after a wildfire, and thus the zones are characterized by the presence of species of the genus *Cistus* (*C. salviifolius* L., *C. monspeliensis* L.). In such conditions, it is obvious that the species can play an important role in the successions of recovery after a wildfire, prior to the beginning of the bushes (*Pistacia lentiscus* L.) stage, or to the germination resumption of the woodland itself. Inside the forests, plants of *S. garganica* develop an impressive growth, with very long procumbent stems. In the same environment, populations of *S. garganica* can be observed along the sides of paved roads (provincial roads), as part of a road’s vegetation (Peschici area). In this case, the plant community *S. garganica* belongs to is usually characterized by *Brachypodium retusum* (Pers.) P.Beauv. There is typically the coexistence on rocky substrates in southern Gargano of plant species characterizing the chasmophytic flora of Gargano [28,39], such as *Lomelosia crenata* (Cirillo) Greuter & Burdet subsp. *dallaportae* (Boiss.) Greuter & Burdet and *Inula verbascifolia* (Willd.) Hausskn. (sites of Monte Sant’Angelo/Manfredonia), or of the latter together with *Centaurea subtilis* Bertol. (Monte Saraceno, Monte Sant’Angelo/Mattinata). Together with *Campanula garganica* Ten. and *Coronilla juncea* L., *Centaurea subtilis* is on the other hand typical of the plant communities of *S. garganica* in the northern areas of the promontory.

### 3.3. Biological Characterisation of Scabiosa garganica

The several analyzed samples confirmed the nature of a scapose hemicryptophyte, with a vegetative phase and a quite long reproductive activity, with floral scapes developing from late spring to the end of autumn. Full flowerings are typical, even during autumn. The lateral ascending rooting shoots help the horizontal growth of the plant, which often takes a circular shape.

### 3.4. Quantitative Morphometric Characters of Scabiosa garganica

The analyzed individuals (Table S1) present the structure of very flashy plants, 23 cm to 100 cm high. Every individual had 1 to 15 scapes; many flower peduncles developed from them with a length between 3 and 40 cm. The number of flower heads per individual ranged from 2 to 146, while the flower heads’ diameters were between 1.5 and 4.3 cm. The plants had many basal leaves, and the cauline ones grew up to one-third of the whole plant. The basal leaves’ length was between 1.07 and 8 cm, and the width between 1.04 and 9.21 cm; the petiole length ranged from 1.02 to 8 cm. The length/width ratio of the leaf was on average 1.22. The cauline leaves on the other hand resulted in being 0.9 to 9.47 cm long, 0.49 to 4.32 cm wide, with a number of lobes between 2 and 16. In every cauline leaf, it was always possible to distinguish an apical segment whose length ranged between 0.58 and 5.76 cm, and whose width was between 0.09 and 3.36 cm; the length/width ratio of the apical segment was on average 2.3. The lateral segments resulted in being long, between 0.21 and 2.25 cm, and wide between 0.14 and 4.65 cm, with a ratio of 3.45. Moreover, the apical segment width/lateral segment width ratio was 4.85.

### 3.5. Qualitative Characters of Scabiosa garganica

The individuals had a very ramified thick root system, from whose base many leafy scapes grew. The leaves had a green/greyish velvety indumentum with patent whitish hairs on both their lower and upper pages. Hairs characterized scapes and flower peduncles. The difference between the cauline and the basal leaves was pronounced: The first ones were opposite and laciniate, imparipinnatesect with a crenate edge, generally linear towards the higher point of the scape; the basal leaves were simple and elliptic-spathulate (generally with rounded apex). The inflorescence had herbaceous bracts on two rows and wrapping a hairy receptacle with bracteoles. The corolla was usually blueish, close to lilac and purplish. The central (pentamerous) flowers were actinomorphic, and the peripheral ones were zygomorphic. The calyx had bristles and setae; each flower had an eight-furrowed tubular involucel. All the four stamens were bilobed.

### 3.6. Typification of the Name Scabiosa garganica

The name *Scabiosa garganica* appears to be still untypified [24]. Given that a type is essential for the correct interpretation of the name, we here proceeded with the designation of the type.

The species was firstly correctly described by Wettstein [15], who described it indicating “Monte Gargano in Italien” (Mt. Gargano in Italy) and referring to the specimens collected by Porta and Rigo, hosted in the herbarium by P. Porta [6]. According to Stafleu and Cowan [40], the Porta collections are preserved in more than thirty herbaria. We were able to trace original material for the name *S. garganica* in BM, FI, JE, K, and TR. All the specimens fit the protologue, in particular those of the *Itinere II Italico*, given that Wettstein reports that the specimens were marked with the number 72 and corresponded to the current taxonomic circumscription of *S. garganica*. According to all data stated, we here designated the specimen preserved in FI barcode FI065250 (left-hand specimen) as a lectotype of the name *Scabiosa garganica*.

***Scabiosa garganica*** Porta & Rigo ex Wettst. 1892: 67(–68, 97, pl. 4, figs. 1–2)

**Type** (lectotype, designated here): ITALY. *Ex itinere II italico*, Italia austral. Apulia: Gargano in pasc. saxos. apricis ad montem St. Angelo, sol. calcar., 600–2000’, 3 July 1875, Porta et Rigo 72 (FI barcode FI065250 left-hand specimen [digital image!], available online at http://parlatore.msn.unifi.it/types/search.php; isolectotypes: BM barcode BM001134486 [digital image!], FI barcode FI065250 right-hand specimen [digital image!], FI barcode FI065251 [digital image!], FI barcode FI065252 [digital image!], FI barcode FI065253 [digital image!], FI barcode FI065255 [digital image!], FI barcode FI065257 [digital image!], FI barcode FI065258 [digital image!], JE barcode JE00016102 [digital image!], JE barcode JE00016103 [digital image!], K barcode K000762977 [digital image!], K barcode K000762978 [digital image!], K barcode K000762979 [digital image!]).

Further original material traced: ITALY. *Ex itinere I. italico Portae et Rigoi*, Italia austr. Apulia, in pascuis Mt. S. Angelo in Gargano, 1(000)-2000’ s.m., sol. calcar., 4 July 1874, Porta et Rigo s.n. (FI barcode FI065254 [digital image!], FI barcode FI065256 [digital image!], K barcode K000762976 [digital image!]); Apulia, in saxosis apricis Gargani circa M.te S. Angelo, sol. cal., alt 1(000)-2000’, 03/07/1875, Porta s.n. (TR 030714 [digital image!]).

### 3.7. Conservation Status of S. garganica

The conservation status of *S. garganica* was evaluated according to the IUCN categories and criteria [32]. The only criterion that could be applied was criterion B (i.e., geographic range). With an extent of occurrence (EOO) of 970 km^2^, calculated as convex hull; an area of occupancy (AOO) of 52 km^2^, calculated using 2 × 2 km grid cells; a number of locations (sensu IUCN) of six; and an estimated continuing decline of EOO and quality of habitat, mainly due to the construction of buildings and the recovery of the pine forest, we considered the species Vulnerable, VU B1ab(i,iii) + 2ab(i,iii).

### 3.8. Comparison with S. holosericea and S. taygetea

**Figure 2:**
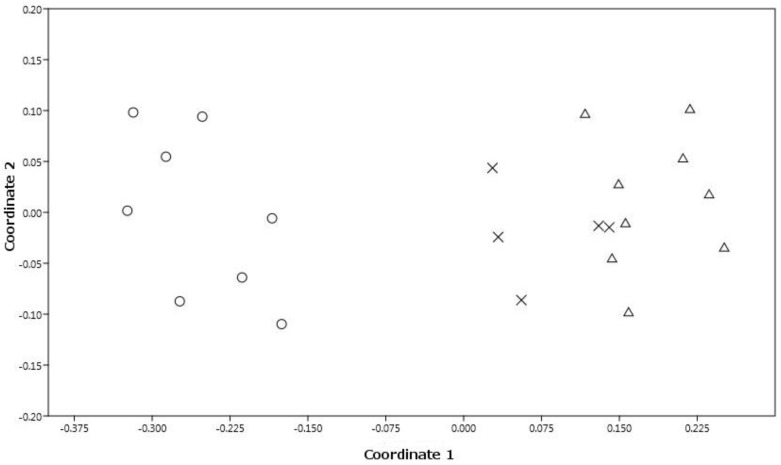
Non—metric multidimensional scaling scatterplot showing the first two dimensions of the analysis. Legend: circle (*S. garganica*), triangle (*S. taygetea*), X (*S. holosericea*).

**Figure 3:**
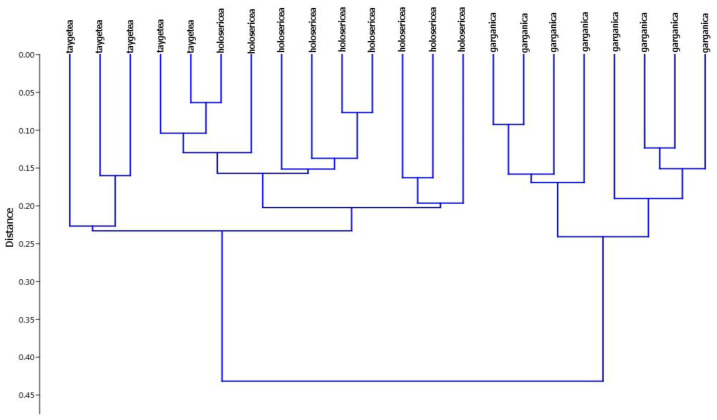
Hierarchical clustering of individuals of *S. garganica*, *S. holosericea,* and S. *taygetea* using a paired group algorithm (UPGMA) and Gower Similarity Index.

**Figure 4:**
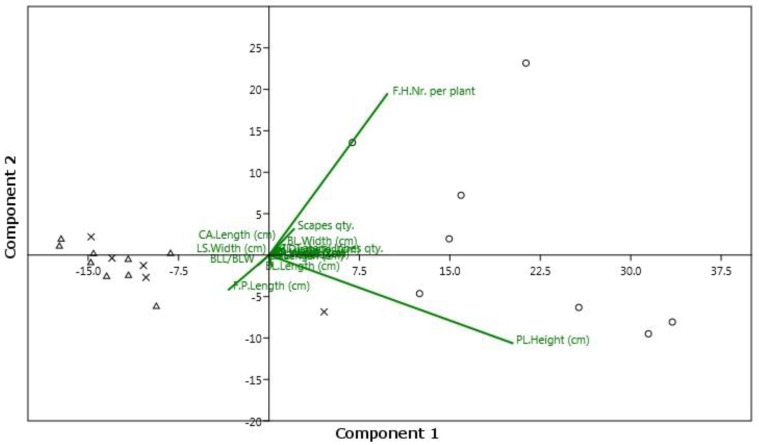
PCA plot. Component1 (25.08%), Component2 (19.55%). Legend: circle (*S. garganica*), triangle (*S. taygetea*), X (*S. holosericea*).

The NMDS analysis, performed with three dimensions, yielded an ordination with a stress value of 0.09122. The scatterplot shows on the first two axes a clear distinction between *S. garganica* and *S. holosericea* and *S. taygetea*, and no overlapping areas among *S. garganica* and the other individuals (Figure 2). The UPGMA dendrogram (Figure 3) yielded two well-defined clusters, one including all individuals of *S. garganica* and the other all individuals of *S. holosericea* and *S. taygetea*. The PCA plot (Figure 4) shows how plant height and the number of flower heads per plant were the most important discriminatory variables between *S. garganica* and *S. holosericea* and *S. taygetea*; furthermore, the PCA plot indicates the clear separation between *S. garganica* and the other taxa, with the samples of the latter to the left and the *S. garganica* samples to the right of axis 1 of the Component1 principal coordinates, representing 25.08% of the total variation of the dataset.

Comparisons of morphological characters between *S. garganica*, *S. holosericea,* and *S. taygetea* are summarized in Table 2.

The comparison between *S. garganica*, *S. holosericea,* and *S. taygetea* shows that *S. garganica* is an independent species, endemic to southern Italy (Gargano and doubtfully occurring in Basilicata).

## 4. Discussion

In a triangular territory whose farther points are Monte Sant’Angelo, Manfredonia, and Mattinata, we found most of the locations reported by Fenaroli [19], i.e., Micheli [7] (ascending Mount Gargano), Trotter and Forti [41] (between Manfredonia and Monte Sant’Angelo), Porta and Rigo [6] (near Monte Sant’Angelo), and Lacaita [17] (400 m a.s.l., Monte Sant’Angelo/Manfredonia road). These stations refer also to two out of the nine studied populations in the present research, located along the road leading from Monte Sant’Angelo to Manfredonia. In this same area, we might recognize Fiori’s station (Valle delle Macchie in Monte Sant’Angelo territory) and Agostini’s station (between Monte Sant’Angelo and Mattinata—collected in 1961 and preserved in Herb. Fenaroli-TR), erroneously attributed by Fenaroli to *Scabiosa holosericea*, a species that is not present in Gargano. In this part of the territory, in fact, are three of our collecting stations, all characterized by the occurrence of *Scabiosa garganica* (without *Scabiosa holosericea*). Moreover, the samples collected at Pulsano Abbey (between Monte Sant’Angelo and Manfredonia) have been attributed by Fenaroli to *S. holosericea*, but they belong to *S. garganica* as well.

Outside this area, the station reported by Martelli in Testa del Gargano (FI!, collected in 1893), a few kilometers far from Vieste (not ascertained by our survey), is interesting and requires more field research to confirm or exclude it from the current distribution of the species.

Our collecting stations in the northern Gargano area significantly expanded the knowledge on the distribution of *Scabiosa garganica* in this part of the promontory, where the species was reported by Licht [29] in Monte Pucci (west of Peschici).

With regard to the distribution, further reflection is needed about the different altitude conditions in northern Gargano stations (exposed to winds from the north/north-east) and the southern ones. A similar disjunction on the Gargano Promontory has been observed in the distribution of another plant species of conservation interest, namely, *Centaurea subtilis* [42]. Such distributional differences between the north and south parts of Gargano can be due to climatic (exposure to the winds from north-east) and/or pedological reasons (different soil’s conditions). In northern Gargano, the bioclimatic zone of *Scabiosa garganica* does not span beyond 100 m a.s.l., while in southern Gargano, it appears until 1000 m a.s.l.; the pedological conditions the plant is found in in northern Gargano starts from 20 m a.s.l., yet it can be found in the southern zone only above 300 m a.s.l.

The analysis performed on the abundant samples collected define important new quantitative parameters that might help to recognize the species today: According to Wettstein [15] and Pignatti [1], the plants’ height is between 30 and 50 cm, but our data reveal a much wider range, i.e., 23 to 100 cm. The number of scapes also was proven to be much greater (four on average), being included in a range between 1 and 15. Apart from the scape number, another difference we noted between our data and those from the literature is the number of flower heads: Pignatti [1] reports a maximum of three flower heads, but plants with up to 146 heads have been found, and they never have a unique flower head. Other interesting data regard the flowering period: According to Porta and Rigo [6], it is in summer, while our survey found flashy and rich blossoming that continued until December, and thus we might say it has a summer/autumn blossoming. Similar as reported in “Flora d’Italia” [1], the color of the corolla is typically purple/blueish.

Lastly, the data collected are somehow enough to clarify the morphological relationship with species close to *Scabiosa garganica*. The comparison highlights significant differences with regard to the flower heads’ number (generally unique in *S. holosericea,* up to 3 in *S. taygetea* and up to 146 in *S. garganica)*, plant height (*S. garganica* is higher), and their indumentum (soft green/yellowish hairs in *S. taygetea*, whitish and thick in *S. holosericea* and *S. garganica*). Other differences can be observed in terms of the color of the corolla, being purple/blueish in *S. garganica* and *S. holosericea*, but different from Tilli’s [9] cerulean/purplish and Wettstein’s [15] red/violet of *S. taygetea*. As for *S. holosericea*, our research also examined wide zones of Gargano Promontory without finding any presence of plants attributable to *S. holosericea,* so that the reports of Pasquale and Licopoli [13], Fenaroli [19], Fanelli et al. [31], and Licht [22] can be attributed to *Scabiosa garganica*, as also indicated by Licht and Wagensommer [25].

According to the IUCN categories and criteria [32], *S. garganica* is Vulnerable. Even if most of the territory occupied by the species is protected by the Gargano National Park, specific conservation actions, both in situ and ex situ, should be implemented in order to ensure the long-term conservation of this rare species endemic to southern Italy.

## 5. Conclusions

Thanks to the research performed in the Gargano area, *Scabiosa garganica* is now a species defined under the morphological aspect, and hence it gets an undeniable autonomy from *S. holosericea* and *S. taygetea*; therefore, the taxon *Scabiosa garganica* Porta & Rigo ex Wettst. can be considered fully valid. Between these taxa, there are a few unquestionable similarities, such as the typical hairs of all the three species; on the other hand, the morphological differences—starting from the color of the corolla and ending with the plant structure—are significant, as demonstrated by the statistical analysis. *S. garganica* can be recognized thanks to its flashy structure (the plant is up to 1 m high and has many scapes), to the large number of flower heads (up to 146), and from its summer/autumn blossoming. Our research also defined the habitat of *S. garganica* (altitude, exposure, nature of the substrate). Even if *S. garganica* should be considered an Italian endemism (Gargano and doubtful in Basilicata), its relationships with *S. taygetea* make it interesting from a phytogeographical point of view. In fact, both if *S. garganica* is considered occurring in Italy and Greece [2] or as endemic to Italy [1], i.e., as a vicariant of the related *S. taygetea* growing in Greece, from a phytogeopraphical point of view, it represents one of the many taxa occurring in the Gargano Promontory and linked to the Balkan flora, such as *Bromus parvispiculatus* H.Scholz [43], *Cerinthe retorta* Sm. [44,45], *Linum elegans* Spruner ex Boiss. [46], and *Ophrys oestrifera* M.Bieb. aggr. [47]. In Italy, all these species are rather rare.

In conclusion, phytosociological surveys in the plant communities where *S. garganica* has been found would be beneficial to better understand the synecological role of this interesting species of the Italian flora.

***Specimina visa* of *Scabiosa holosericea* and *S. taygetea*:**

*Scabiosa holosericea* Bertol.: ITALY. S.l., August 1908, Fiori A. (LY barcode LY0312723); Toscana: Alpes Apuanes à Fornole, July 1862, Savi P. (LY barcode LY0312729); Alpi Apuane, 1867, Bertoloni, A. (K barcode K000762986); Alpi Apuane, 1818, Schleicher, J.C. (K barcode K000762985); Alpi Apuane, M. Altissimo versante settentr., 5 October 1951, Pichi Sermolli R.; van Steenis C.G.G.J., Contardo A.; det. R. Corradi (L barcode L2979831); Calabria: M. Pollino, in pascuis saxosis elatis, solo calc., 1800–2000, 29 July 1898, Rigo G. (LY barcode LY0312718).

*Scabiosa taygetea* Boiss. & Heldr.: Greece. Kalamata, Montes Taygetos, ad semitam inter pagum Tòryza et montem Profictis Elias, 1200–1500 m, in regione abietina, 26 June 1978, Cernoch F. (BR barcodes BR00000027547878V, BR0000025719024V); M. Taygetus, 1885, Haussknecht C. (JE barcode JE00016176); In reg. sylvatica M. Taygeti, July 1844, Heldreich T.H.H. (W barcode WU077546); In rupestris regionum superiorum Taygeti, August 1844, Heldreich T.H.H. (W barcode W0050831).

## Figures and Tables

**Table 1:** Historical reports of *Scabiosa garganica* from Gargano.

Author(s)	Scientific Name Used by the Author(s)
Micheli [7]	*Scabiosa garganica*
Tilli [9]	*Scabiosa garganica*
Pasquale and Licopoli [13]	*Scabiosa holosericea*—*S. taygetea*
Porta and Rigo [6]	*Scabiosa garganica*
Arcangeli [11]	*Scabiosa garganica*
Parlatore and Caruel [12]	*Scabiosa pyrenaica* var. *garganica*
Wettstein [15]	*Scabiosa garganica*
Huter [16]	*Scabiosa holosericea*
Lacaita [17]	*Scabiosa garganica*
Fiori [18]	*Scabiosa garganica*
Fenaroli [19]	*Scabiosa holosericea* subsp. *garganica*
Zangheri [20]	*Scabiosa vestita* subsp. *garganica*
Pignatti [21]	Included in the variability of *Scabiosa holosericea*
Conti et al. [23]	*Scabiosa taygetea* var. *garganica*
Di Pietro & Wagensommer [30]	*Scabiosa taygetea* subsp. *garganica*
Peruzzi et al. [24]	*Scabiosa garganica*
Bartolucci et al. [4]	*Scabiosa taygetea* subsp. *garganica*
Pignatti [1]	*Scabiosa taygetea* subsp. *garganica*
Licht [29]	*Scabiosa taygetea* subsp. *garganica*
Licht & Wagensommer [25]	*Scabiosa taygetea* subsp. *garganica*

**Table 2:** Quantitative characters, reported as mean ± standard deviation, minimum and maximum (extreme values in brackets).

	*S. holosericea*	*S. garganica*	*S. taygetea*
Plant height (cm)	(26.54) 31.72 ± 3.84 (37.79)	(23) 59.24 ± 18.77 (100)	(15.31) 34 ± 10.6 (49.79)
No. of inflorescences	(1) 1.37 ± 0.51 (2)	(2) 18.59 ± 23 (146)	(1)1.71 ± 0.75 (3)
Inflorescence diameter (cm)	(0.61) 1.42 ± 0.61 (2.47)	(1.5) 2.79 ± 0.45 (4.3)	(0.7) 1.41 ± 0.7 (2.44)
Flower peduncles (cm)	(14.74) 21.55 ± 5.41 (31.35)	(3) 14.18 ± 6.39 (40)	(3.24) 15.84 ± 8.68 (30.83)
No. of scapes	(1) 1.33 ± 0.5 (2)	(1) 4.18 ± 3.33 (15)	(1) 1.71 ± 0.75 (3)
BASAL LEAVES			
Lamina length (cm)	(2.25) 3.48 ± 0.71 (4.93)	(1.07) 3.49 ± 1.47 (8)	(2.25) 3.26 ± 0.77 (4.77)
Width (cm)	(0.64) 1.22 ± 0.33 (1.77)	(1.04) 3.31 ± 1.26 (9.21)	(0.96) 1.42 ± 0.34 (1.86)
Petiole length (cm)	(1.38) 1.94 ± 0.45 (2.82)	(1.02) 3.32 ± 1.29 (8)	(1.39) 2.83 ± 1.76 (5.90)
Lamina length/Width (average)	3	1.22	2.32
CAULINE LEAVES			
*Apical segment*			
Length (cm)	(0.99) 2.12 ± 0.83 (3.22)	(0.58) 2.82 ± 1.03 (5.76)	(1.73) 2.15 ± 0.58 (3.24)
Width (cm)	(0.23) 0.77 ± 0.39 (1.31)	(0.09) 1.48 ± 0.67 (3.36)	(0.83) 1.06 ± 0.18 (1.25)
Length/width (average)	3.11	2.3	2.03
*Lateral segment*			
Length (cm)	(0.39) 0.88 ± 0.34 (1.22)	(0.21) 1.09 ± 0.38 (2.25)	(0.57) 0.83 ± 0.16 (1.02)
Width (cm)	(0.14) 0.28 ± 0.12 (0.41)	(0.14) 0.41 ± 0.37 (4.65)	(0.14) 0.28 ± 0.12 (0.41)
Length/width (average)	3.16	3.45	3.02
*Total leaf*			
Length (cm)	(2.62) 2.96 ± 0.34 (3.34)	(0.9) 4.75 ± 1.52 (9.47)	(2.26) 3.22 ± 0.77 (4.32)
Width (cm)	(0.14) 0.28 ± 0.12 (0.41)	(0.49) 2.21 ± 0.73 (4.32)	(1.02) 1.47 ± 0.23 (1.65)
No. of leaf lobes	(2) 4.4 ± 1.51 (6)	(2) 6.69 ± 2.78 (16)	(4) 5 ± 1.09 (6)
Apical segment width/lateral segment width (average)	2.7	4.85	2.19

## Data Availability

Data is contained in the article, including the Supplementary Material.

## Supplementary Materials

The following supporting information can be downloaded at https://www.mdpi.com/article/10.3390/plants12091915/s1, Table S1: Collecting sites of *Scabiosa garganica* samples used for the present research. Table S2: Dataset composed of all the measurements carried out on *Scabiosa garganica*, *S. holosericea,* and *S. taygetea*.
